# Steps towards the synthetic biology of polyketide biosynthesis

**DOI:** 10.1111/1574-6968.12365

**Published:** 2014-01-07

**Authors:** Matthew Cummings, Rainer Breitling, Eriko Takano

**Affiliations:** 1 Faculty of Life Sciences, Manchester Institute of Biotechnology, The University of ManchesterManchester, UK

**Keywords:** refactoring, plug-and-play biology, combinatorial biosynthesis, secondary metabolites, drug discovery

## Abstract

Nature is providing a bountiful pool of valuable secondary metabolites, many of which possess therapeutic properties. However, the discovery of new bioactive secondary metabolites is slowing down, at a time when the rise of multidrug-resistant pathogens and the realization of acute and long-term side effects of widely used drugs lead to an urgent need for new therapeutic agents. Approaches such as synthetic biology are promising to deliver a much-needed boost to secondary metabolite drug development through plug-and-play optimized hosts and refactoring novel or cryptic bacterial gene clusters. Here, we discuss this prospect focusing on one comprehensively studied class of clinically relevant bioactive molecules, the polyketides. Extensive efforts towards optimization and derivatization of compounds via combinatorial biosynthesis and classical engineering have elucidated the modularity, flexibility and promiscuity of polyketide biosynthetic enzymes. Hence, a synthetic biology approach can build upon a solid basis of guidelines and principles, while providing a new perspective towards the discovery and generation of novel and new-to-nature compounds. We discuss the lessons learned from the classical engineering of polyketide synthases and indicate their importance when attempting to engineer biosynthetic pathways using synthetic biology approaches for the introduction of novelty and overexpression of products in a controllable manner.

## Polyketides: magnificently modular

Polyketides represent an important class of compounds that are extremely diverse in structure and function. Natural screening strategies have brought more than 20 drugs to market, including the immunosuppressants FK506 and rapamycin (Park *et al*., 2010; Goranovic *et al*., 2012); hypocholesterolemics, such as lovastatin (Ma & Tang, 2007); anticancer agents, such as doxorubicin (Vasanthakumar *et al*., 2013); and a host of antimicrobials, including tetracycline and erythromycin (Weissman & Leadlay, 2005; Lesnik *et al*., 2009). Furthermore, it is predicted that more than 1% of polyketides described have potential drug activity (Koskinen & Karisalmi, 2005), and as a result of this, polyketides have received tremendous attention in efforts to unearth new compounds with bioactive properties. Interest in the discovery of novel acting polyketides has been renewed with the recent surge in microbial genome sequences and the availability of accurate genome mining software to detect a previously unexpected abundance of uncharacterized secondary metabolite biosynthesis gene clusters (BGCs). Advances in sequence analysis software such as antiSMASH are providing a facility to screen for BGCs in an automated computational fashion (Medema *et al*., 2011a, b2011b; Blin *et al*., 2013). They are particularly powerful in detecting polyketide BGCs, as these are defined by the presence of highly characteristic signature genes and motifs. Identification of putative BGCs using sequence-based analysis is also enabling the discovery of compounds that are cryptic, which are not expressed under laboratory conditions.

In addition to the clinical relevance and abundance of polyketides, there is one other reason behind the particular interest in polyketides as promising targets for synthetic biology: the highly modular architecture of both the BGCs and the constituent polyketide synthases (PKS) presents an ideal starting point from which to engineer chemical novelty in polyketides.

**Figure 1:**
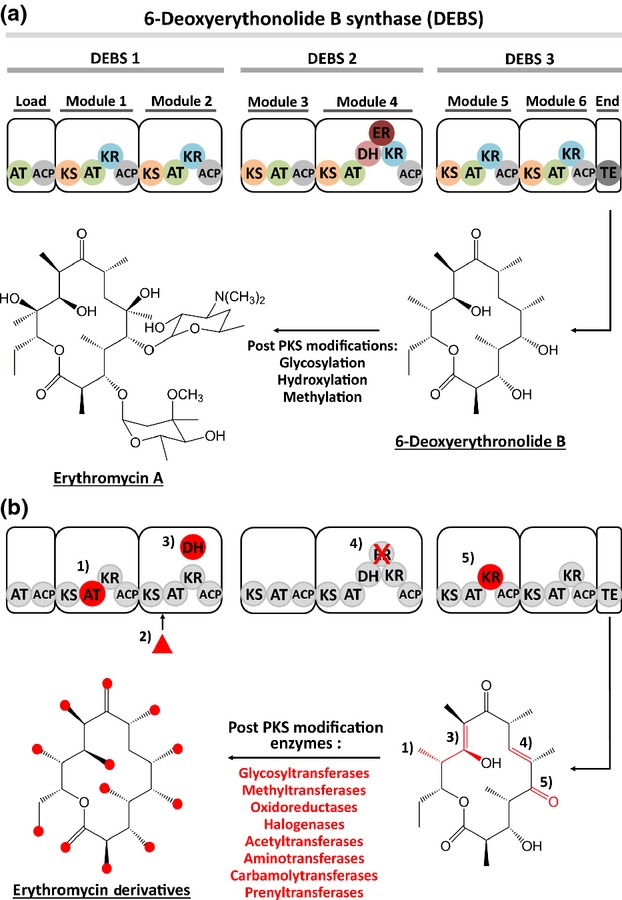
Pictorial illustration of 6-DEBS synthase, a modular type I PKS and successful attempts at engineering this megasynthase. (a) The native biosynthetic gene cluster and end product. (b) Summary of engineered cluster variants and their products; alterations are indicated in red. Manipulation of the polyketide scaffold includes: (1) substitution of domains (Oliynyk *et al*., 1996); (2) feeding with noncanonical substrates (Jacobsen *et al*., 1997); (3) domain insertion (McDaniel *et al*., 1997); (4) inactivation of domains (Donadio *et al*., 1993); and (5) domain deletions (Donadio *et al*., 1991). The effects of modifications 1–5 to the 6-dEB scaffold are also indicated in red, as are the positions at which engineered post-PKS tailoring modifications can occur. AT, acetyltransferase domain; ACP, acyl carrier protein domain; KS, ketosynthase domain; ER, enoyl reductase domain; DH, dehydratase domain.

The biosynthesis of polyketides is modular at many levels. First, the genes responsible for polyketide biosynthesis are typically clustered in the genome (Chen *et al*., 2006), forming a BGC. Each BGC encodes the PKS responsible for the formation of the carbon backbone, together with the tailoring enzymes required for primary tailoring events, for example cyclization and dimerization of the β-keto-acyl carbon chain, subsequent tailoring events to form the final polyketide structure as well as genes encoding the regulation of the BGC and resistance to the end product if applicable, for example in the case of antibiotic end products. Once transcribed and translated, the PKS enzymes themselves are also modular in nature. The best-characterized PKS, 6-deoxyerythronolide B (6-dEB) synthase from *Saccharopolyspora erythraea* (Mironov *et al*., 2004), represents a good example of this. 6-dEB synthase consists of three megasynthases encoded by three ORFs, DEBS1–3 (Fig.1a). Each of these megasynthases comprises a series of modules responsible for the extension of the polyketide carbon backbone through addition and selective reduction of one acyl-CoA monomer to form a β-keto-acyl intermediate. In addition to this, each module can be dissected further still into a series of domains. Each of these domains is unequivocally linked with one specific catalytic function required for chain extension. Some domains are obligatory for recruitment of the acyl-CoA monomer and chain extension, for example acetyltransferase, acyl carrier protein (ACP) and ketosynthase, while others are accessory domains involved in the selective reduction of the β-keto-acyl intermediate to the corresponding alcohol, olefin or methylene group catalysed by ketoreductase, dehydratase and enoyl reductase activity, respectively. Importantly, all of the modules encoded within DEBS1–3 are required for successful synthesis of 6-dEB and act in succession, like a giant molecular assembly line (Weissman & Leadlay, 2005). Because each domain is unequivocally linked with one specific catalytic function and polyketides are synthesized in a collinear fashion, addition, removal and/or substitution of these domains or modules will theoretically result in defined alterations of the end product. Furthermore, the collinear architecture of these domains, and motifs within, can allow prediction of the structure of the polyketide and important elements of its stereochemistry from analysis of its coding sequence (Caffrey, 2003; Reid *et al*., 2003; Anand & Mohanty, 2012). With these rules in mind, theoretically, we have the potential to engineer rationally a desirable predefined polyketide end product if domains or modules can be stitched together like molecular lego bricks (Weber *et al*., 2003; Kennedy, 2008).

With the increase in the number of characterized PKSs, it is becoming apparent that the collinear relationship between gene structure and chemical end product is not absolute (Piel, 2010); however, as a general rule, collinearity presents an ideal template for engineering the polyketide biosynthetic machinery. Consequently, manipulating polyketide assembly through domain alteration has been one major avenue that classical engineering has explored in order to derivatize known polyketides even before the current era of synthetic biology (Fig.1b; Donadio *et al*., 1991, 1993; Kao *et al*., 1994, 1995; Oliynyk *et al*., 1996; McDaniel *et al*., 1997; Ranganathan *et al*., 1999; Rowe *et al*., 2001). However, not all PKSs show the ‘one domain–one reaction step’ modular organization that is seen in type I PKSs. Chain extension can also occur iteratively through a recursive approach, where domains that are part of a single polypeptide are used repeatedly. This is the case for type II and type III PKSs, as well as for some type I fungal PKS, for example LovB and LovC in lovastatin biosynthesis (Campbell & Vederas, 2010). Although differences occur in enzymatic organization of PKSs, the underlying chemistry behind chain extension, through successive decarboxylative Claisen condensation of acyl-CoA monomers to form a β-keto-acyl intermediate and modification in *cis* or *trans*, remains the same for all (Lai *et al*., 2006). As such, all PKSs are in principle amenable to engineering (Kantola *et al*., 2003; Yu *et al*., 2012). Detailed reviews of the underlying biochemistry are available (Lai *et al*., 2006; Hertweck, 2009; Meier & Burkart, 2009; Walsh & Fischbach, 2010; Williams, 2013).

The immense diversity in the chemical structures of polyketides is the result of continuous evolutionary pressure for the development of chemical novelty facilitated by the modular nature of the PKS. On an evolutionary scale, diversity is introduced into polyketides through both simple mutations within domains and frequent horizontal co-transfer of genes between clusters (Donadio *et al*., 2005). Evolutionary analysis reveals conserved synteny between gene clusters responsible for the biosynthesis of homologous products, as well as products of considerable structural difference and those in between. Transfer of gene units between BGCs, permitted by their inherent modularity and collinearity, generates a continuous interspecific flow of compounds with novel physicochemical properties, not only as polyketides, but also for the generation of hybrid products containing additional nonribosomal peptide moieties. The recently described BGC encoding the biosynthesis of three zeamine-related antibiotics in the *Serratia plymuthica* RVH1 genome provides a good example of the plasticity of BGCs and their ability to co-transfer between organisms. This BGC comprises genes for five PKSs, three nonribosomal peptide synthetases and one mixed fatty acid synthase/PKS enzyme, which are required for the synthesis of the hybrid product backbones, as well as additional tailoring genes (Masschelein *et al*., 2013). Hybrid products, such as these, elucidate the tolerance of synthases to integrate noncanonical substrates from different biosynthetic systems into the growing carbon backbone successfully and are naturally occurring versions of domain alteration attempts paralleled in the laboratory-based engineering of PKSs.

Generation of novelty through exchange of domains between BGCs polished under evolutionary selection pressures, as above, invariably results in successful product assembly – as millions of failed ‘experiments’ are rapidly discarded by natural selection. This process cannot be replicated easily *in vitro*, and simple domain substitutions between BGCs commonly result in the failure of product release and maturation (Xu *et al*., 2013). Failure of product biosynthesis is regularly the result of the inflexibility of downstream enzymes to tolerate novel substrates. Without additional engineering, most domains incorporate only one substrate into the growing polyketide backbone and show little flexibility to introduce noncanonical substrates. Lessons learned from reprogramming PKSs using classical molecular biology approaches, detailed below, are supporting this general observation, but also, more interestingly, are revealing exceptions. This has provided an instruction manual that exemplifies the scope and limitations of plasticity of PKS to tolerate the integration of exogenous extenders into the growing β-keto-acyl chain.

**Figure 2:**
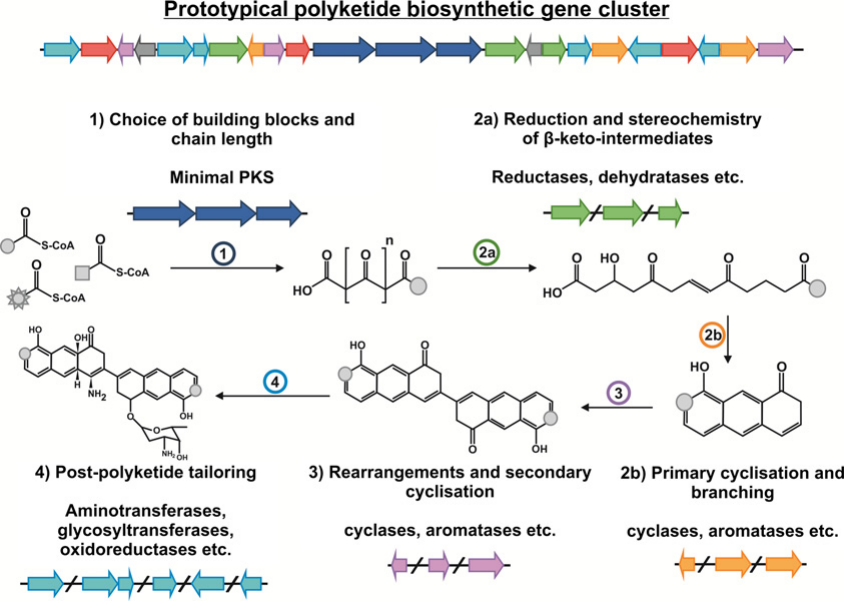
Schematic illustration of the four steps of polyketide biosynthesis encoded by a prototypical polyketide biosynthetic gene cluster. Each of these steps offers the potential for end product diversification by evolution or engineering as described in the text.

Diversification of polyketides can occur at four steps throughout biosynthesis resulting from: (1) the choice of building blocks and chain length, (2) the extent of reduction and stereochemistry of β-keto intermediates (Reid *et al*., 2003), primary cyclization, alkylation and branching, (3) rearrangements and secondary cyclization and (4) postpolyketide tailoring: glycosylation, oxygenation, etc. In the following discussion, we focus on these events as two main phases of polyketide synthesis: core scaffold biosynthesis (steps 1–3) and subsequent or concurrent tailoring events (step 4; Fig.2).

## Modularity of scaffold biosynthesis

### Initiation of biosynthesis

The composition of the polyketide backbone, or scaffold, structure is governed by the stringency of acetyltransferase domains to load a specific acyl-CoA substrate, but also through substrate stereochemistry and redox pattern (Sundermann *et al*., 2013): each PKS assembles an individual product through the choice of acyl-CoA units, their level of reduction and subsequent tailoring. Initiation of scaffold biosynthesis requires selection and recruitment of a starter unit onto a didomain, comprising an acetyltransferase and an ACP, collectively termed the loading module. The resulting initial starter unit serves as the first substrate in the growth of the final β-keto-acyl chain. Generation of diversity through the promiscuity of acetyltransferase domains to load multiple different starter units, termed polyspecificity, is more commonly observed than by polyspecificity of extender modules later in biosynthesis (McDaniel *et al*., 1999; Yuzawa *et al*., 2012). Introduction of diversity during initiation of biosynthesis also commonly occurs through the multiple different priming mechanisms used by the array of loading modules available (Moore & Hertweck, 2002; Hertweck, 2009). Due to the mechanistic promiscuity of the starter domains, combinatorial biosynthesis attempts to manipulate PKS modules often start with here. For example, the acetyltransferase and ACP loading module of DEBS1 naturally recruit a propionate starter unit. Substitution with loading modules from tylosin and oleandomycin type I megasynthases from *Streptomyces fradiae* and *Streptomyces antibioticus*, respectively, resulted in controlled integration of propionate or acetate as a starter unit (Long *et al*., 2002). Similarly, the replacement of the isobutyryl-CoA-specific loading module initiating avermectin biosynthesis in *Streptomyces avermitilis* M1 by the unique phoslactomycin polyketide cyclohexanecarboxylic unit loading module from *Streptomyces platensis* resulted in production of the veterinary antiparasitic doramectin (Wang *et al*., 2011). Alteration of loading modules for the initiation of biosynthesis is therefore one step showing promise for the generation of novel polyketides.

### Chain extension

After initiation, continued assembly of the polyketide scaffold requires loading of extender units onto the acetyltransferase and ACP and incorporation into the β-keto-acyl intermediate by the ketosynthase. At this stage, diversity can be introduced through the installation of noncanonical extender units resulting from the polyspecificity of loading domains, domain substitutions or by the iterative action of an otherwise modular PKS (Kapur *et al*., 2012). The collection of commonly used extender units nature provides is modest: Canonical extender units comprise malonyl- and methylmalonyl-CoA. Substitution for domains loading other, less commonly used, extender units will allow introduction of a broadened chemistry into the polyketide backbone. For example, reductive carboxylation of α,β-unsaturated acyl-CoA precursors via crotonyl-CoA reductase/carboxylase homologues facilitates inclusion of hexyl-, propyl-, chloroethyl- and isobutylmalonyl-CoA into the polyketide scaffold (Eustaquio *et al*., 2009; Liu *et al*., 2009; Wilson *et al*., 2011). Alternatively, functionalization of extender units on stand-alone ACPs allows the incorporation of allyl- (Mo *et al*., 2011), amino-, hydroxyl- (Chan *et al*., 2006) and methoxymalonyl-ACP (Wu *et al*., 2000) extender units into the polyketide scaffold.

Predicting the polyspecificity of extender modules to introduce these rarer extender units is not straightforward. The mechanical processing and discrimination between acyl-CoA extender units by loading domains in type I modular PKSs is currently little understood. Investigations to elucidate why particular substrates are preferred or chosen are presenting a growing body of evidence suggesting that PKSs may be able to tolerate and incorporate exogenous natural and non-natural extender units into the β-keto-acyl chain. For example, analysis of the acyl-CoA substrate selectivity of PikAIV, a pikromycin synthase from *Streptomyces venezuelae*, elucidated the polyspecificity of extender modules towards substrates not readily present in the producer. PikAIV successfully loaded malonyl-, propionyl-, ethyl- and native methylmalonyl-CoA to the ACP. In the case of malonyl- and propionyl-CoA, active site occupancy was low at 3% and 19%, respectively. More interestingly, the rare extender ethylmalonyl-CoA showed acetyltransferase loading of 90% and low levels of hydrolytic release indicating its potential for incorporation during assembly; the native substrate methylmalonyl-CoA showed 100% acetyltransferase saturation (Bonnett *et al*., 2011). In the case of PikAIV, all acyl-CoA substrates were loaded; however, incorporation into the carbon chain depended upon the rate of subsequent hydrolytic release. These findings suggest that extender modules may show a greater tolerance to incorporate exogenous precursors lacking evolved selectivity, consistent with findings previously reported (Pohl *et al*., 2001). Substituting extender domains for the addition of novel extender units showing limited hydrolytic release could therefore result in the generation of novel polyketides; however, no concrete rules defining what properties extender modules require to do so have been elucidated, despite observed discrimination between sizes of extender units and incorporation.

### Product release

Manipulating starter and extender modules of PKSs may permit the introduction of novel acyl-CoA substrates into the polyketide scaffold. However, for these to show activity, they must be released from the PKS. For successful release, it is important to identify which catalytic domains act as decision gates, thereby permitting continuation of downstream biosynthesis of altered β-keto-acyl intermediates. Elucidating such points will significantly aid success when engineering BGCs. Yeh *et al*. (2013) experimentally indicated that the phylogeny between nonreducing iterative PKS (nrPKS) modules is a good predictor of successful polyketide assembly and release from engineered BGCs. Increased phylogenetic proximity between gene units translated to improved domain–domain interactions and as a result, improved the release of the polyketide end product. In contrast, Xu *et al*. (2013) show for type II nrPKSs that the best predictors of thioesterase acceptance, and therefore release, are the shape and size of the polyketide substrate and consequently indicate the stringency of thioesterase domains in carrying out discriminative decision gate functions. For example, if the native substrate of a thioesterase was a nonaketide, but the engineered assembly line presented it with a heptaketide, the rate of release was almost zero (Vagstad *et al*., 2013). Substituting thioesterase domains often resulted in abolished product formation, despite the presence of an abundance of β-keto-acyl intermediates produced by the upstream domains, whereas judicious choices of thioesterase substitution resulted in the successful production of an unnatural polyketide product, radilarin (Xu *et al*., 2013). Successful polyketide release from thioesterase in the case of resorcyclic acid lactones and dihydroxyphenylacetate acid lactones may be dependent upon substrate size (Xu *et al*., 2013). However, contrastingly, truncation of the DEBS1–3 megasynthase through relocation of the thioesterase domains downstream of the modular DEBS1 resulted in assembly and successful release of a much shortened triketide lactone (Kao *et al*., 1995; Pfeifer *et al*., 2001). These contrasting results indicated the complex nature of the thioesterase and show the requirement for further work to build rules to predict thioesterase domain tolerance for substrates. Currently, for successful incorporation of novel starter and extender units, and successful product release, analysis of domains must be carried out on a case-by-case basis.

## Modularity of tailoring reactions

Introducing diversity within the polyketide scaffold provides the ability to diversify the backbone structure. Further tailoring of these structures generates an additional level of complexity, and pathway engineering over the past decade has generated new-to-nature products through novel glycosylation, acyltransfer, hydroxylation, epoxidation, alkylation, transamination and desaturation reactions acting on naturally occurring products (Rix *et al*., 2002; Olano *et al*., 2010).

Tailoring enzymes can introduce chemical groups that often are more relevant to engineer for the alteration of specific activity of the polyketide than the backbone construct. 6-dEB is a precursor in the biosynthesis of the macrolide antibiotic erythromycin. Biosynthesis of erythromycin requires the action of tailoring enzymes encoded by ORFs located within the BGC encoding DEBS1–3. Without the required glycosylation, hydroxylation and methylation reactions catalysed by tailoring enzymes, 6-dEB cannot become active as erythromycin (Weissman & Leadlay, 2005). This is similarly the case for a group of type II aromatic polyketides with anticancer activities, the anthracyclines. The mechanisms of action of anthracyclines such as doxorubicin are mediated through DNA damage caused by the inhibition of DNA topoisomerase II, DNA binding and subsequent alkylation and intercalation within DNA of the target cells (Minotti *et al*., 2004). While the basic aglycone structures comprise 7,8,9,10-tetrahydro-5,12-naphthacene quinones, the observed anticancer activities of anthracyclines are heavily dependent on the attached sugars (Weymouth-Wilson, 1997). Furthermore, alteration of the attached sugars can modify not only activity, but also other parameters, such as toxicity. The clinical applications of doxorubicin are limited by dose-dependent cardiotoxic side effects. Epirubicin, an analogue of doxorubicin, with opposing configuration of a C-4 hydroxyl group on the deoxysugar, shows significantly less cardiotoxicity, while maintaining comparable antitumor properties (Hurteloup & Ganzina, 1986). Therefore, exchanging the sugars attached to the aglycone scaffold can tune the overall properties of therapeutic polyketides. Derivatization in this manner could be achieved through the addition of glycosyltransferases into BGCs, as opposed to introducing the sugar moieties semi-synthetically. First steps towards this have been undertaken (Han *et al*., 2011) and are revealing the substrate tolerance of individual tailoring enzymes.

In a parallel to the promiscuity of the scaffold biosynthesis genes, a case study of glycosyltransferases showcases the ability of tailoring enzymes to accept a broader range of substrates and a tolerance to modifying foreign acceptors molecules. ElmGT from *Streptomyces olivaceus* involved in elloramycin biosynthesis (Ramos *et al*., 2008) can glycosylate 8-demethyltetracenomycin C with D-mycarose, D-olivose, L-olivose, L-rhodinose, L-rhamnose and a disaccharide comprising two D-olivose moieties, showing extensive tolerance to glycosylate scaffolds with multiple sugars. Other glycosylases show tolerance to introduce a more defined range of sugars to a wider number of acceptor scaffolds. For example, the L-olivosyl glycosyltransferase OleG2 from *S. antibioticus* involved in oleandomycin production has promiscuity for NDP-L-mycarose, NDP-L-rhamnose and the foreign acceptor erythronolide B. The activities of ElmGT and OleG2 show a high tolerance to introduce noncanonical substrates onto aglycone scaffolds. This level of promiscuity for novel substrates and scaffolds is also consistent for EryCIII and EryBV from *S. erythraea* endogenous to the erythromycin BGC and the heterologously expressed UrdGT2 from *S. fradiae* Tü2717 involved in urdamycin A production, for the generation of novel C-glycosylated compounds (Wohlert *et al*., 1998; Doumith *et al*., 1999; Aguirrezabalaga *et al*., 2000; Gaisser *et al*., 2000; Rodriguez *et al*., 2000; Tang & McDaniel, 2001; Yoon *et al*., 2002).

Such flexibility of tailoring modifications may be a result of frequent co-transfer of tailoring genes between clusters, or a result of multifunctional tailoring of a series of different polyketides within the host; however, in both cases, the ability to introduce a variety of scaffolds opens up promising prospects for further diversification by synthetic biology. Full characterization and cataloguing of tailoring enzymes in a standardized ‘biobrick’ fashion would ultimately allow a user to pick and choose which modifications are desirable (Knight, 2003). Integration of tailoring enzymes into plug-and-play hosts as well as in standardized constructs would facilitate the rapid derivatization of novel compounds. Tailoring in this fashion shows potential to speed up rational design of polyketides at the increased rate that will be necessary, for example, to overcome the rapid and unavoidable emergence of resistance against them in pathogenic bacteria.

## Synthetic biology: the future of combinatorial biosynthesis

Combinatorial biosynthesis has successfully exploited the functional collinearity of PKS domains, their structural modularity across many levels and their enzymatic flexibility and promiscuity for noncanonical substrates, to expand the accessible part of the polyketide universe. Progress, however, has been slower than expected. This is predominantly a result of our inability to predict the tolerance of enzymes to facilitate the downstream biosynthesis or incorporation of novel substrates. The inherent complexity of PKSs may exceed our capacity to define a set of pre-established rules that when followed ensure a judicious choice of modules or domains incorporated into a reengineered BGC for the successful generation of novel products. To overcome this limitation, high-throughput approaches, on a scale comparable to the working of evolutionary recombination, will be necessary. This is where synthetic biology's recent advances of writing genetic code at an unprecedented scale and complexity will usefully complement the repertoire of classical genetic engineering methodologies.

The first and most obvious application of synthetic biology will be for the production of novel compounds discovered by genome mining and metagenomics. It has been generally observed that the majority of BGCs in newly sequenced genomes are cryptic or silent, and the corresponding products are not produced at detectable levels in normal culture conditions. This is usually due to strict repressive control of gene expression by global regulation embedded within the coding sequence in the form of a complex combination of promoters, 5′-UTRs (Breaker, 2008; Ishihama, 2010), feed-forward and feed-back loops (Vasanthakumar *et al*., 2013), pause sites and small noncoding RNA (Georg & Hess, 2011; Guell *et al*., 2011). The intertwined nature of the control circuitry makes it difficult to circumvent native regulation and force expression. ‘Refactoring’, a synthetic biology methodology derived from software engineering, aims at decoupling this endogenous regulation through a comprehensive rewriting of BGCs. The resulting DNA sequence of a refactored BGC is as dissimilar as possible from the wild-type DNA sequence, yet still encoding the same amino acid end products. Rewriting the BGC in this fashion will remove all internal redundancy and regulation, including those regulatory elements that are currently undiscovered (Temme *et al*., 2012). Once a gene cluster has been refactored, we are able to introduce new, controllable and desired regulation to allow biosynthesis and characterization of the end compound, for example using orthogonal T7-based promoter libraries (Alper *et al*., 2005; Shis & Bennett, 2013).

Refactoring BGCs aims to by-pass the classical discovery limitations, decoupling desired product expression from the complex endogenous regulatory cascade. But the resulting engineered clusters can also be designed in such a way that they facilitate further reengineering through additions, deletions, substitutions, domain swapping or other modifications (Fig.1b). Of course, the same limitations for successful product assembly and release apply as they do in combinatorial biosynthesis. This also implies that the generation of novel products cannot reliably be achieved using random modularization of gene units from a multitude of different sources. Refactoring, however, is providing the methodology required to generate vast libraries of BGCs for the prospective biosynthesis of novel PKSs, and by its highly parallel approach, it may enable the elucidation of more informative assembly rules for the engineering of chemical novelty.

Refactoring approaches to synthetic biology do not need to be restricted to the nucleotide level. Ultimately, the aim would also be to optimize the encoded protein sequences to enhance their modularity and thus increase the engineering potential of PKSs. Advances towards this are already being made (Lockless & Muir, 2009); however, as a general strategy, such protein-level refactoring is currently still unrealistic. Nonetheless, the deluge of new genome sequence data is providing increasingly detailed insights into the rules that govern domain compatibility during the natural evolution of polyketide diversity (Chen *et al*., 2007; Thattai *et al*., 2007; Yuzawa *et al*., 2012), and multiplexed genome engineering strategies (Wang & Church, 2011) can be used to systematically explore these rules in the context of specific biosynthetic pathways.

In conclusion, the engineering-inspired approach of synthetic biology raises the dissection, standardization and decoupling of distinct catalytic units from highly integrated cellular processes to new levels of ambition. By learning from the pioneering efforts of combinatorial biosynthesis, as described above, these emergent technologies will soon yield the raw materials required to construct rationally designed biosynthetic machinery and regulatory circuits from first principles on a scale and, at a speed, far superseding our current capacity. Ultimately, the success of the next generation of polyketide bioprospecting for drug discovery will depend on an intimate interaction between protein chemistry, evolutionary genomics and synthetic biology.
